# The protocol for the Cannabidiol in children with refractory epileptic encephalopathy (CARE-E) study: a phase 1 dosage escalation study

**DOI:** 10.1186/s12887-018-1191-y

**Published:** 2018-07-07

**Authors:** Darren Reithmeier, Richard Tang-Wai, Blair Seifert, Andrew W. Lyon, Jane Alcorn, Bryan Acton, Scott Corley, Erin Prosser-Loose, Darrell D. Mousseau, Hyun J. Lim, Jose Tellez-Zenteno, Linda Huh, Edward Leung, Lionel Carmant, Richard J. Huntsman

**Affiliations:** 1 Cannabinoid Research Initiative of Saskatchewan (CRIS), University of Saskatchewan, Saskatoon, Saskatchewan Canada; 2 College of Pharmacy and Nutrition, University of Saskatchewan, Room E3210 Health Sciences 104 Clinic Place, Saskatoon, SK S7N-2Z4 Canada; 3 Department of Pediatrics, Division of Child Neurology, Loma Linda University, Loma Linda, California, USA; 4 Division of Pediatric Neurology, Department of Pediatrics, University of Alberta, 11405-87 Avenue, 4th Floor, Edmonton, AB T6G-1C9 Canada; 5 Department of Pharmaceutical Services, Saskatchewan Health Authority, Saskatoon Health Region, Royal University Hospital, 103 Hospital Drive, Saskatoon, SK S7N-0W8 Canada; 6 Department of Pathology and Laboratory Medicine, Saskatchewan Health Authority, St. Paul’s Hospital, 1702 20th Street West, Saskatoon, SK S7M-0Z9 Canada; 7 Saskatchewan Health Authority and Department of Psychology, University of Saskatchewan, Saskatoon, Saskatchewan Canada; 8 Department of Clinical Health Psychology, Royal University Hospital, 103 Hospital Drive, Saskatoon, SK S7N 0W8 Canada; 9 Clinical Trial Support Unit, University of Saskatchewan, Royal University Hospital, Room 5676, 103 Hospital Drive, Saskatoon, SK S7N 0W8 Canada; 10 Department of Pediatrics, University of Saskatchewan, Royal University Hospital, Room 2665, 103 Hospital Drive, Saskatoon, SK S7N 0W8 Canada; 11 Cell Signalling Laboratory, Departments of Psychiatry and Physiology, University of Saskatchewan, GB41, HSB 107 Wiggins Ave, Saskatoon, SK S7N 5E5 Canada; 12 Department of Community Health and Epidemiology, University of Saskatchewan, Room E3222 Health Sciences, 104 Clinic Place, Saskatoon, SK S7N-2Z4 Canada; 13 Department of Medicine, Division of Neurology, University of Saskatchewan, Royal University Hospital, Room 1622, 103 Hospital Drive, Saskatoon, SK S7N 0W8 Canada; 14 Division of Pediatric Neurology, Department of Pediatrics, University of British Columbia, BC Children’s Hospital, Room 2D19, 4480 Oak Street, Vancouver, BC V6H-3V4 Canada; 15 Division of Pediatric Neurology, Room CE208, Department of Pediatrics 5, University of Manitoba, Children’s Hospital, 840 Sherbrooke Street, Winnipeg, MB R3A-1S1 Canada; 16 Division of Pediatric Neurology, Department of Pediatrics, Centre Hospitalier Universitaire Sainte-Justine, Universite de Montreal, Room 5-4, 3175 Chemin de la Cote Ste-Catherine, Montreal, QC H3T-1C5 Canada; 17 Department of Pediatrics, Royal University Hospital, Rm 2744, 103 Hospital Drive, Saskatoon, SK S7N 0W8 Canada

**Keywords:** *Cannabis*, Cannabidiol, Pediatric epilepsy, CanniMed®

## Abstract

**Background:**

Initial studies suggest pharmaceutical grade cannabidiol (CBD) can reduce the frequency of convulsive seizures and lead to improvements in quality of life in children affected by epileptic encephalopathies. With limited access to pharmaceutical CBD, *Cannabis* extracts in oil are becoming increasingly available. Physicians show reluctance to recommend *Cannabis* extracts given the lack of high quality safety data especially regarding the potential for harm caused by other cannabinoids, such as Δ^9^-tetrahydrocannabinol (Δ^9^-THC). The primary aims of the study presented in this protocol are (i) To determine whether CBD enriched *Cannabis* extract is safe and well-tolerated for pediatric patients with refractory epilepsy, (ii) To monitor the effects of CBD-enriched *Cannabis* extract on the frequency and duration of seizure types and on quality of life.

**Methods:**

Twenty-eight children with treatment resistant epileptic encephalopathy ranging in age from 1 to 10 years will be recruited in four Canadian cities into an open-label, dose-escalation phase 1 trial. The primary objectives for the study are (i) To determine if the CBD-enriched *Cannabis* herbal extract is safe and well-tolerated for pediatric patients with treatment resistant epileptic encephalopathy and (ii) To determine the effect of CBD-enriched *Cannabis* herbal extract on the frequency and duration of seizures. Secondary objectives include (i) To determine if CBD-enriched *Cannabis* herbal extracts alter steady-state levels of co-administered anticonvulsant medications. (ii) To assess the relation between dose escalation and quality of life measures, (iii) To determine the relation between dose escalation and steady state trough levels of bioactive cannabinoids. (iv) To determine the relation between dose escalation and incidence of adverse effects.

**Discussion:**

This paper describes the study design of a phase 1 trial of CBD-enriched *Cannabis* herbal extract in children with treatment-resistant epileptic encephalopathy. This study will provide the first high quality analysis of safety of CBD-enriched *Cannabis* herbal extract in pediatric patients in relation to dosage and pharmacokinetics of the active cannabinoids.

**Trial registration:**

http://clinicaltrials.gov [Internet]. Bethesda (MD): National Library of Medicine (US). 2016 Dec 16. Identifier NCT03024827, Cannabidiol in Children with Refractory Epileptic Encephalopathy: CARE-E; 2017 Jan 19 [cited 2017 Oct]; Available from: http://clinicaltrials.gov/ct2/show/NCT03024827

## Background

The epileptic encephalopathies are a group of childhood-onset seizure disorders characterized by frequent seizures and markedly abnormal EEG patterns associated with progressive disturbance of cerebral function that manifests as developmental stagnation or regression. These epilepsies are often resistant to conventional medical treatment regimens and children with these conditions invariably experience neurological and cognitive impairments that severely impair their quality of life (QoL) [1].

In 2013 Porter and Jacobson reported the results of a 24-point survey they posted on a *Facebook*-group composed of parents using CBD-enriched *Cannabis* products to treat their children with refractory epilepsy. Of the 20 respondents, 84% reported the CBD-enriched *Cannabis* products resulted in a decrease in seizure frequency in their children and over half of their children either became seizure-free or had a greater than 80% reduction in their seizure frequency. Just as importantly, most parents reported an improvement in QoL indices such as alertness, sleep, and mood [2]. Since that time several open-label and randomized double-blind trials of CBD-based treatments in children with epileptic encephalopathy including Dravet Syndrome and Lennox Gastaut syndrome have been reported [3–6]. These studies found a reduced frequency of convulsive seizures and mild adverse events of somnolence and elevated liver-enzyme activities. Unfortunately, there was considerable variation in the dosage and types of CBD formulation used; three studies using a purified CBD product (Epidiolex) and one using a whole plant *Cannabis* herbal extract. The considerable variation in CBD dosage and lack of pharmacokinetic data resulted in no guidance on appropriate dosage regimens in this pediatric patient population.

CBD can be derived from pure pharmaceutical preparations or in extracts of *Cannabis sativa* or *Cannabis indica* [7]. The composition of *Cannabis* extracts can vary dramatically due to differences in cultivars, growing conditions, and extraction and decarboxylation processes. The lack of standardization or quality assurance in the preparation and dose administration of these products severely limits the scientific study of herbal preparations of *Cannabis*. The recent availability of commercial *Cannabis* extracts from a licensed medical marijuana producer that uses good manufacturing processes (GMP) with assayed cannabinoid composition assures patient safety and reliable dosing and enables scientific evaluation [8, 9]. We propose to conduct an open-label dose escalation study of CBD-enriched *Cannabis* herbal extract in pediatric patients with treatment resistant epileptic encephalopathy.

## Methods/Design

### Objectives

#### The primary objectives of the CARE-E study are:

1. To determine if a CBD-enriched *Cannabis* herbal extract is safe and well-tolerated for pediatric patients with treatment resistant epileptic encephalopathy.
2. To monitor the effects of a CBD-enriched *Cannabis* herbal extract on the frequency and duration of specific seizure types.

#### Secondary Objectives

1. To determine whether CBD-enriched *Cannabis* herbal extract will alter steady-state levels of co-administered anticonvulsant medications.
2. To assess how treatment of pediatric patients with treatment refractory epileptic encephalopathy with CBD-enriched *Cannabis* herbal extract will affect the patient’s QoL.
3. To determine the relation between dose escalation and steady-state trough levels of bioactive cannabinoids.
4. To determine the relation between dose escalation and improvement in seizure frequency, QoL and incidence of adverse effects.

##### Study product

The study product is an oil-based extract of *Cannabis sativa* purchased from CanniMed® Therapeutics Incorporated (Saskatoon, Canada) named ‘CanniMed® Oil 1:20’ with 1 mg/mL of Δ^9^-THC and 20 mg/mL of CBD. CanniMed® operates under the *Access to Cannabis for Medical Purposes Regulations* governed by Health Canada [10] using GMP. The general process for harvest, ethanol extraction, decarboxylation, concentration and solution in olive oil is described by CanniMed® [11]. The concentrations of Δ^9^-THC and CBD in the product, and lack of mold, mycotoxins, and pesticides are confirmed by a third party laboratory as mandated by Health Canada. The product is purchased as 60 mL graduated amber oval bottles (PETE) that are sealed with child-proof caps, labeled according to local law and identified by the protocol number and dosage. The Research Pharmacy at each site will receive the study product from CanniMed® for subsequent distribution to their site’s participants. As an oil-based suspension the product will be taken orally or by gastrostomy tube and the volume varies according to the weight of the participant. A single lot number of product was provided by CanniMed® for this study to ensure consistency of dosing. The product was purchased from CanniMed® at cost and this research remained independent of the company by securing all funding through external research grants.

##### *Study population*

The study will recruit participants between the ages of 1–10 years with an epileptic encephalopathy resistant to standard medical treatment. The study will aim to enroll 28 children from four Canadian cities (anticipated seven participants per site).

##### *Study design*

The CARE-E trial is a phase 1, open-label, dose-escalation study consisting of 4 separate phases: recruitment, baseline, treatment, and weaning. The recruitment phase involves the selection of eligible participants using pre-established exclusion and inclusion criteria (described below). The baseline phase establishes baseline values for each experimental measurement prior to treatment with the study product. During the treatment phase, caregivers of participants administer dosages of the CBD-enriched *Cannabis* herbal extract twice daily to their children escalating at fixed one-month intervals over the course of four-months. Upon completion of the treatment phase, participants will enter the weaning phase and caregivers will slowly taper the participants off of the CBD-enriched *Cannabis* herbal extract using a one-month weaning schedule.

**Fig. 1:**
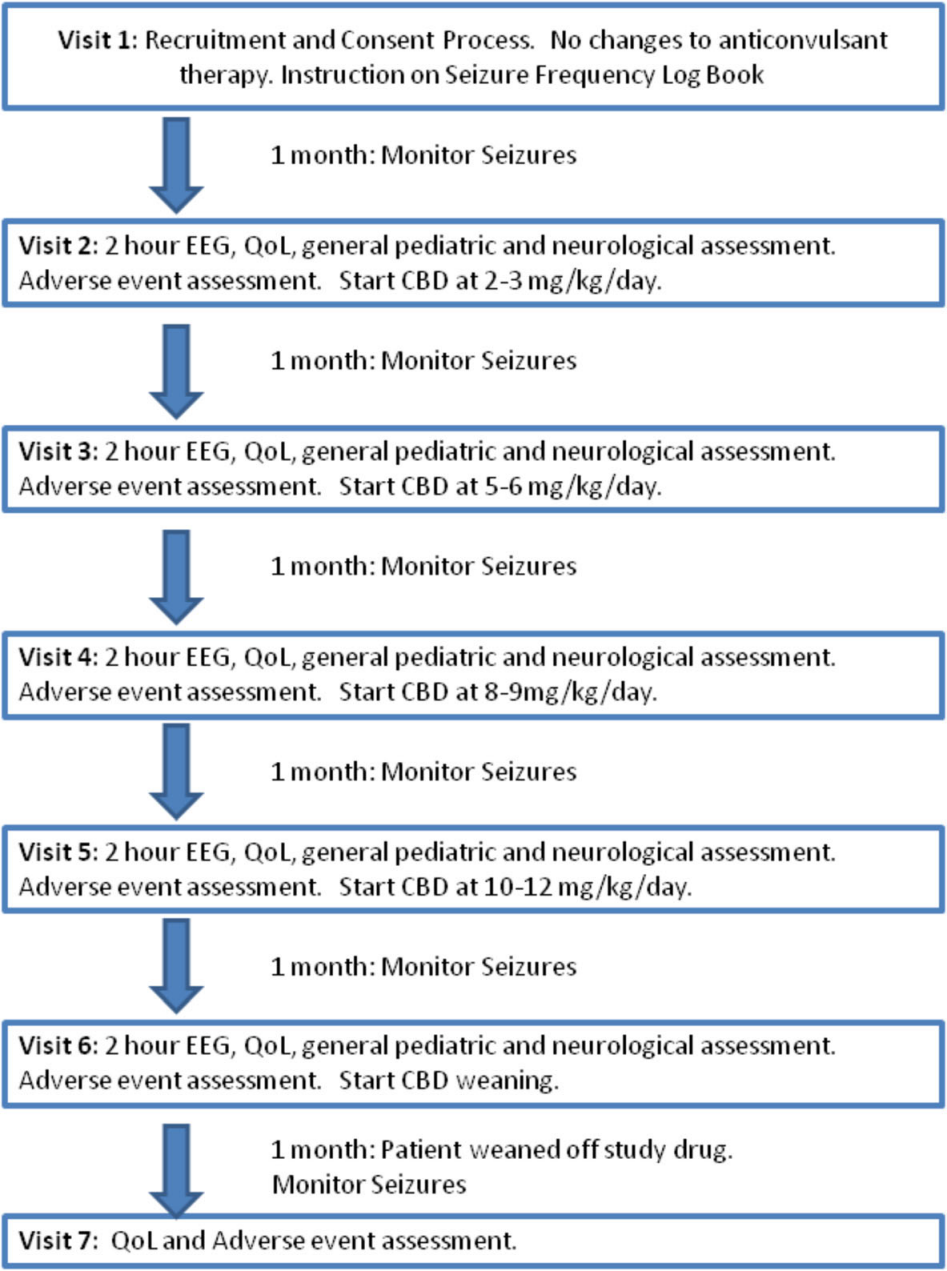
A flow chart of participant enrollment, treatment with CBD-Enriched *Cannabis* herbal extract, monitoring and weaning

During the study, caregivers will monitor the participants for any potential side effects and will use a study diary to record their child’s seizure activity by tracking seizure frequency and duration, and any use of rescue medications to abort prolonged seizures. The participant’s condition as well as drug levels and biomarkers of toxicity will be monitored on a monthly basis. Testing will include blood and urine analysis, QoL assessments, neurological and general pediatric assessments, and an electroencephalogram (EEG) recorded for 2 h or until sleep is obtained (Fig. 1).

Recruitment Phase: Prospective participants will be directly identified and recruited through the caregivers by study physicians at each study site. Any potential participants’ caregiver will be contacted by the study physician or pediatric neurology nurse either in-person at the study physician’s clinic or by telephone. Prospective caregivers of participants will be asked if they are interested in having their child participate in the study. If the response is positive, a copy of the study brochure and consent form will be provided to them. Caregivers of prospective participants will be asked to attend a recruitment visit after they agree to participate in the study and provide informed consent. During the recruitment visit, the participant will be screened for eligibility based on specific inclusion and exclusion criteria. If the participant qualifies for the study, the participants’ caregivers will be instructed on use the study diary.

Inclusion and exclusion criteria: Participation in this study is inherent on meeting the following inclusion criteria: (1) Participants must be between the ages of 1 and 10 years of age with treatment-resistant epileptic encephalopathy including: Infantile Spasms, Continuous Spike Wave in Sleep, Lennox Gastaut, Doose, Landau-Kleffner and Dravet Syndromes and Malignant Migrating Partial Seizures of Infancy. ‘*Treatment-resistant’* will be in keeping with the International League Against Epilepsy (ILAE) definition of failing two appropriate anticonvulsant medications at therapeutic doses. (2) Participants must experience a minimum of at least one major seizure *per* week or four major seizures *per* month. For the purposes of this study, major seizures will be motor seizures including: atonic, tonic, clonic, tonic-clonic, major myoclonic, myoclonic astatic seizures and epileptic spasms. (3) Participants must be available to attend study assessments regularly and enter data into the seizure monitoring logs correctly. (4) Negative pregnancy test at screening for females who have reached menarche.

Exclusion criteria for the study include (1) Recent (< 1 month) change in anticonvulsant therapies including anticonvulsant medications, ketogenic diet or settings on Vagal Nerve Stimulator (VNS) (2) Recent (< 6 months) change in intravenous immunoglobulin (IVIG) treatment. (3) Initiation of ketogenic diet within 6 months of study enrollment. (4) Implantation and activation of VNS within 12 months of study enrollment. (5) Use of cannabis-based therapy within two months of study enrollment. (6) Use of a Selective Serotonin Reuptake Inhibitor (SSRI), a tricyclic antidepressant, or an atypical neuroleptic in the month prior to study enrollment. (7) Concomitant regular concomitant use of narcotics (use of narcotics in emergency situations under the supervision of a physician is allowed). (8) Initiation or dosage change in oral or injectable steroid therapy within three months of study enrollment. (9) Allergy or known intolerance to any ingredient in the study compound. (10) Inability to attend assessments on a monthly basis. (11) Clinically significant cardiac, renal or hepatic disease (as assessed by the site investigator).

Subject Withdrawal Criteria: A participant may be withdrawn from the study if: (1) The study drug is causing intolerable side effects or a worsening in the participant’s seizures; (2) The caregiver fails to give the study drug to the participant as prescribed; (3) The caregiver does not bring the participant to appointments; (4) The study at a particular site is cancelled by the principal investigator, a site investigator or the institutional sponsor for administrative or other reasons. Whenever possible, the participant withdrawn from the study will continue to receive a dosage schedule that gradually weans the participant off the study drug over a one-month period. However, if the site investigator deems it medically necessary for the participants’ safety, the participant could be weaned off the study drug faster. All participants that complete the study will be asked to return for an end of study visit (Visit 7). All data collected about the participant during enrolment will be retained for analysis and the participant will not be replaced.

Baseline Phase: Following the recruitment visit, participants will be sent home for one month with no change to their current anticonvulsant therapy, ketogenic diet, or Vagal Nerve Stimulator settings. Caregivers will be asked to track their child’s seizure frequency, duration, and use of rescue medication during this month. Rescue medications allowed for home-use include: Ativan (0.1–0.2 mg/kg PRN intrabucally, sublingual or IV), Midazolam (0.1–0.2 mg/kg PRN intranasally, intrabucally or IV), or Diazepam (0.2–0.5 mg/kg PRN rectally or IV). Other rescue medications may be administered by paramedics (under physician guidance) or physicians as *per* hospital guidelines or the child’s individual guidelines for management of *status epilepticus*. At the end of this month, participants and their caregivers will be required to visit the study clinic for a series of baseline tests including: blood and urine analyses, quality of life and cognitive/developmental assessments, neurological and general pediatric assessment, and an EEG lasting 2 h or until the participant falls asleep. Data from the seizure diaries will be collected and a new diary will be provided for the following month.

Treatment phase: Initiation of therapy: Following baseline testing, caregivers of participants will receive a 33-day supply of the 1:20 Δ^9^-THC:CBD *Cannabis* herbal extract from the site research pharmacist at visit 2. Caregivers of participants will be instructed to administer the study product at a 1:20 Δ^9^-THC:CBD *Cannabis* herbal extract dose of 2–3 mg/kg/day divided into two doses (BID). Caregivers will be further instructed to monitor their child’s seizure activity as defined above. In addition, they will be asked to monitor their child for any potential side effects such as drowsiness, ataxia, nausea, vomiting, worsening seizures, etc.

Monthly follow-up: Caregivers will return to the clinic for the monthly testing as described above. Data from the study diaries will be copied for analysis. Following the completion of testing, parents will receive a new 33-day supply of the 1:20 THC:CBD *Cannabis* herbal extract from the research pharmacist. Parents will be instructed to administer the extract at increasing doses over the next 3 months; i.e. at 5–6 mg/kg/day divided BID at visit 3, 8–10 mg/kg/day divided BID at visit 4, and 10–12 mg/kg/day divided BID at visit 5. If the participant experiences significant side-effects at a certain dose, the subsequent CBD dose will be adjusted to the mid-point between their current dose and former dose. Parents will be instructed to continue tracking their child’s seizure activity and monitoring the child for potential side effects in the same manner as the initiation of therapy month.

### Dosage of 1:20 Δ^9^-THC:CBD *Cannabis* herbal extract

#### Rationale for escalating dose of CBD to 10–12 mg/kg/day

As there is no available pediatric pharmacokinetic data for the cannabinoids including CBD and THC, the dosage regimen used in this study is extrapolated from CBD dosages previously described in the literature [2–6]. Consideration is made of the fact that the study product is derived from a whole plant extract that contains Δ^9^-THC among other potentially biologically active cannabinoids and terpines.

In Jacobson and Porter’s report, most children who had a positive response to CBD were taking a dose ranging from 8 to 14 mg/kg/day [2]. Devinsky and Thiele used a dose of 20 mg/kg/day in their participants randomized to receive study drug but this was a purified CBD product with negligible concentrations of Δ^9^-THC [5, 6]. Tzadok’s study participants received a CBD dose of either < 10 mg/kg/day or 10–20 mg/kg/day provided in the form a CBD-enriched *Cannabis* extract [4].

#### Regarding calculation of dosage and distribution of 1:20 Δ^9^-THC:CBD Cannabis herbal extract at each study visit

To ensure consistency between centers in the dosing regimen for their study participants, for each dosing increment for the participant, the mid-point value of the dosage range be chosen and the daily dosage be rounded to the nearest 10 mg CBD (0.5 ml of *Cannabis* Extract). This will also allow for greater ease and accuracy in administering the study drug to the participants by their caregivers. For example, a participant who weighs 25 kg at Visit 1 would be prescribed a daily dose of 60 mg CBD (2.4 mg/kg/day) to commence on Visit 2. The dosage for each visit would be calculated on the preceding visit to allow time for the site’s research pharmacy to order the study drug so it can be delivered on time by the producer.

### Drug distribution and accountability

In order to comply with Health Canada requirements for a clinical study involving a *Cannabis* product, care is taken to ensure accountability with regards to the amount of 1:20 Δ^9^-THC:CBD *Cannabis* herbal extract dispensed to- and utilized by- the study participant. Proper disposal of unused or excess *Cannabis* herbal extract must be ensured. For this reason, the *Cannabis* herbal extract will be distributed via the research pharmacies at each study site. This will allow for greater accountability with regards to the amount of *Cannabis* herbal extract dispensed to and used by the study participants. This will also prevent the possibility of *Cannabis* herbal extract being shipped to participants who have withdrawn from the study or fail to attend study visits. As a total supply for 33 days will be allotted to each participant to allow some flexibility in scheduling study visits, Health Canada Section 56A Exemptions had to be obtained for the research pharmacy at each study site. Upon receipt of the 1:20 Δ^9^-THC:CBD *Cannabis* herbal extract by the research pharmacy, the quantity received will be recorded in a drug receipt record and the 1:20 Δ^9^-THC:CBD *Cannabis* herbal extract will be stored in a locked drug cabinet at the research pharmacy until such time that it will be dispensed to the participant. Once dispensed by the research pharmacy to the participant, the amount dispensed as well as the date and time will be recorded in a drug dispensing log. When the study participant returns for their subsequent visit, they will return all empty bottles as well as any unused 1:20 Δ^9^-THC:CBD *Cannabis* herbal extract to the research pharmacy. The amount of 1:20 Δ^9^-THC:CBD *Cannabis* herbal extract returned will be recorded in the drug dispensing log and a calculation will be performed to ensure it matches the estimated amount that should have been returned based on the participant’s daily dose and the date of return. To help contain costs of performing this study, for visits 3–6, any unused 1:20 Δ^9^-THC:CBD *Cannabis* herbal extract will be re-dispensed to the study participant and calculated into the total amount dispensed. At visit 7, any unused 1:20 Δ^9^-THC:CBD *Cannabis* herbal extract will be recorded and stored along with the unused 1:20 Δ^9^-THC:CBD *Cannabis* herbal extract for all participants at that site to be destroyed as per the research pharmacy’s specific guidelines.

Weaning phase: Termination of treatment: At visit 6 (after completing 1 month of CBD at 10–12 mg/kg/day) participants will return to the clinic for a final series of tests which include: blood and urine analyses, quality of life and cognitive/developmental assessments, neurological and general pediatric assessments, and EEG. Participants will be provided with a one-month weaning schedule which incrementally decreases the dose of the 1:20 Δ^9^-THC:CBD *Cannabis* herbal extract administered (CBD at 8–9 mg/kg/day for 1 week then 5–6 mg/kg/day for 1 week then 2–3 mg/kg/day for 1 week prior to discontinuing the study product).

Final Assessment: Participants will return to the clinic upon completion of the one-month weaning period. Caregivers will provide observations of any side-effects noted during the weaning period and will complete a final quality of life questionnaire. Data from the seizure monitoring diaries will be collected and caregivers will be asked to return any leftover study drug.

### Experimental measurements

#### Bioactive cannabinoid plasma concentrations

A secondary study objective is to determine the relationship between dose escalation and steady state trough concentrations of bioactive cannabinoids, and if possible, relate these levels with therapeutic and adverse effects. To achieve this objective a liquid chromatography-mass spectrometry (LC-MS/MS) method was validated in accordance with the United States FDA guidelines [12, 13]. Blood collected into lithium heparin Barricor vacutainers ® (BD Canada, Mississauga, ON) at each visit will be centrifuged (10 min at 1500 rpm), the plasma aliquoted into clearly labeled microcentrifuge tubes, and placed at − 80 °C until analysis. Plasma concentrations of THC, CBD and THC-OH (11-hydroxy-THC) in participant plasma samples will be determined by LC-MS/MS analysis. Briefly, stock solutions (1 mg mL^− 1^) of cannabinoids and their respective stable isotope labeled internal standards (Cerilliant Corp., Round Rock, TX) will be prepared in methanol and stored at − 20 °C. Working solutions will be prepared by serial dilution of the stock solution in blank human plasma to produce appropriate standard calibration curves. Acceptance criteria for each analytical run will be based on low, medium, and high concentration quality control (QC) standards. Calibration and QC samples will be prepared on each day of sample analysis. A linear least-squares regression analysis using 1/X^2^ as weighting factor will be conducted to determine the linearity of the calibration curve. Plasma sample extraction involves the addition of 10 μL of the internal standard working solution and 600 μL of cold acetonitrile to 200 μL plasma, followed by vortex-mixing and centrifugation at 20,000 *g* for 10 min at 4 °C. 700 μL of supernatant is dried under filtered air for 15 min at 37 °C. Samples are reconstituted using 200 μL mobile phase. Supernatant will be transferred to HPLC inserts and 5 μL injected onto a Zorbax Eclipse XDB-C18 narrow bore 2.1 × 12.5 mm 5 μm guard column and Zorbax Eclipse XDB-C8 narrow bore 2.1 × 12.5 mm 5 μm guard column with column temperature maintained at 30 °C. The cannabinoids are separated using an Agilent series 1290 binary pump (Agilent Technologies, Mississauga, ON, Canada) with an online degasser and auto sampler set at 4° and a mobile phase of 80% methanol and 20% Solution B (0.1 mM ammonium formate) at a flow rate of 250 μL/min. Injections will occur at 13.5 min intervals and will include linear gradients to 90% methanol 10% Solution B at 3.5 min to 10 min and return to 80% methanol: 20% Solution B from 10 min to 10.5 min.

The cannabinoids will be detected with an ABSciex 6500 QTRAP mass spectrometer (AB Sciex, Concord, ON, Canada) in positive ion mode. Multiple reaction monitoring (MRM) will be used to quantify the cannabinoids and the peak areas will be summed through use of MultiQuant 3.0.1 Software. The ratio of peak areas of the cannabinoids to their respective internal standards will be plotted against the nominal concentrations to construct the calibration curve and the concentrations of the cannabinoids determined by interpolation.

#### Complete blood counts and clinical chemistry

At each visit participants will have laboratory assessment of blood components to evaluate hepatic, renal, or hematopoietic toxicity performed at their local hospital laboratory. The tests performed include: a complete blood cell count panel with automated three or five part cell differential, electrolytes, glucose, creatinine, urea, alanine transaminase, aspartate transaminase, albumin, gamma glutamyl transferase and lipase. Adverse events from each participant will be assessed as laboratory results that exceed the local laboratory age-specific reference intervals. If participants are on a ketogenic diet during the study, then urine ketone testing will be performed to assess the consistency of the ketosis at each visit.

#### Trough levels of anticonvulsants

Participants will remain on pre-existing anticonvulsant medications throughout the cannabis oil study period. Serum specimens will be collected from participants at each visit and trough levels of serum anticonvulsant medications will be determined by LC-MS/MS by the Roy Romano Provincial Laboratory Regina, SK, Canada. Serum specimens were collected and stored at − 20 °C prior to analysis. Adverse events will be counted if participants require a change in anticonvulsant medication during the trial either to maintain trough levels in the therapeutic range.

#### Quality of life assessment

The instrument we have chosen is the Quality of Life in Childhood Epilepsy (QOLCE-55) [14]. The QOLCE is a parent/proxy-completed measure of health-related quality of life specifically developed for children with epilepsy. It has several subsections containing multiple items, as well as a series of global ratings. The original tool was designed for individuals between 4 and 18 years of age which is one of the broadest age ranges for a tool of this kind. The tool allows for the rater to indicate that an item is not applicable if its content is above the age or developmental level of the child being rated. This makes the QOLCE potentially robust in the face of issues such as lower age and intellectual disability.

The QOLCE-55 shows good internal consistency and criterion-related validity as well as adequate to good test-retest reliability, depending on the subtest or item involved [14–16]. Areas covered include physical features (including physical limitations and fatigue), well-being (including depression, anxiety, helplessness and self-esteem), cognition (including attention, memory, language and general cognition), social engagement (including interactions, activities and stigma), and behavior. The QOLCE has also been shown to be sensitive to seizure activity and other clinical and psychosocial variables associated with epilepsy [14] and to benefits from treatments such as surgery [17]. Finally, the QOLCE has been used in the study of epileptic conditions with associated cognitive delays and Intellectual Disability and has already shown its utility in samples with Intellectual Disabilities [18]. While the QOLCE-55 was not exclusively positive in the wording of its items, most items were positively stated, making for less distress on the side of those completing the measure [19].

Ratings on the QOLCE are made on a 5-point scale with 1 titled “very often” and 5 titled “never.” Reversed items are recoded when scoring such that higher scores mean more positive outcomes. These scores are then recoded as follows: 1 = 0, 2 = 25, 3 = 50, 4 = 75, and 5 = 100. The mean for each of the subscales is then found by adding these values together and dividing by the number of items not marked Not Appropriate. The total score for the scale is the unweighted mean of the four subscales.

As well, for the purposes of our study we added 13 additional items based on reports from parents. Additional items covered sleep (including being drowsy), verbal and nonverbal communication, use of books, awareness of surroundings, interpersonal interactions with children and adults, and irritability. These additional items are scored as other QOLCE items and are summed into their own total score as well as being looked at individually.

#### Seizure monitoring

Seizure monitoring will be used to determine how treatment with the study compound affects seizure frequency duration. Caregivers will be asked to track the frequency and duration of their child’s three most frequent types of seizures on a daily basis using a study diary. In order for the study to remain consistent, the caregivers will track the same three types of seizures throughout the study. Seizures that occur in a cluster will be counted as one seizure although the duration of the cluster and number of seizures per cluster will be recorded. Although dialeptic seizures are not included as part of the inclusion criteria for the study, caregivers will be encouraged to record the frequency of dialeptic seizures if their child experienced them frequently.

#### Use of rescue medication

Caregivers will be asked to track their child’s use of rescue medication. This will determine whether treatment with the study compound has any influence on use of rescue medication. Caregivers will record the medication used, the dosage used, and the number of times it was administered.

### Sample size determination

As CARE-E is a phase I dose escalation safety and tolerability study designed to find the most appropriate dose of CBD in a pediatric population it was felt that power analysis was not required to calculate sample size. The sample size of 28 participants each receiving 4 separate dosage escalations is within usual guidelines for standard phase I clinical trial designs. In this multi-site dose escalation study, we chose to escalate within the same participant with 7 participants at each site because the low pediatric population incidence of epileptic encephalopathy (the inclusion criterion), precluded ability to escalate in cohorts of 6, where a new cohort of six would be administered the next dosing level [20, 21]. Any patient exhibiting a dose limiting toxicity will not receive the next dose escalation.

### Data analysis

Study data will be collected and managed using REDCap electronic data capture tools hosted at the University of Saskatchewan [22]. REDCap (Research Electronic Data Capture) is a secure, web-based application designed to support data capture for research studies, providing 1) an intuitive interface for validated data entry; 2) audit trails for tracking data manipulation and export procedures; 3) automated export procedures for seamless data downloads to common statistical packages; and 4) procedures for importing data from external sources.

#### Statistical analysis

All data will be descriptively analyzed using means, standard deviations, frequencies (where appropriate), and 95% confidence intervals. The sample size of 28 participants is sufficient for an initial phase 1 safety and tolerability study, but is too small for precise estimation of steady state levels of biologically active cannabinoids at each dose and for definitive assessments of efficacy. Trends will be examined and a medical statistician will assist with statistical and trend analysis of the data. Complete, specific details of the statistical analysis will be described and fully documented in the Statistical Analysis Plan (SAP) after completion of data collection.

#### Study funding:

Given the potential controversy surrounding the study of *Cannabis* products in children, CARE-E was funded entirely through external funding in order to minimize the potential for perceived bias in our study results. Funding was obtained through research grants from the Jim Pattison Children’s Hospital Foundation (formerly the Children’s Hospital Foundation of Saskatchewan), the Saskatchewan Health Research Foundation and the Savoy Foundation as well as a donation from the Durwood Seafoot Estate (administered through the Jim Pattison Children’s Hospital Foundation).

## Discussion

Children with epileptic encephalopathies resistant to standard therapy are at considerable risk for long-term neurocognitive impairment and poor quality of life. CBD-enriched *Cannabis* based therapies have been shown in several studies to provide a reduction in seizure frequencies and improvements in sleep patterns, mood, and alertness. Such favorable reports in the medical literature and social media have prompted parents who are desperate to help their children to combine *Cannabis* products with current medical treatments in children with refractory epilepsy. However, the encouraging publicity surrounding medical marijuana is not accompanied by strong scientific and rigorous investigation. This is particularly true for this vulnerable pediatric population.

As a Phase I dose escalation study, the CARE-E study is primarily designed to assess safety of a high CBD, low ∆^9^-THC *Cannabis* oil preparation. However, it is anticipated that the study can begin to address other major issues associated with *Cannabis* use in pediatric epileptic encephalopathies, namely the lack of an accepted dosage regimen, the relationship between steady state plasma concentrations and efficacy or adverse effects, its efficacy to reduce seizure frequency and improve quality of life, and potential drug-drug interactions with standard medical treatments for pediatric epilepsy. Successful implementation of the CARE-E study will lay foundation for a larger Phase II efficacy trial of a high CBD, low ∆^9^-THC *Cannabis* oil product. Such studies are imperative to alleviate the lack of clinical information on medical *Cannabis* in children with refractory seizures and give practitioners confidence to prescribe *Cannabis*-derived products to their patients.

While CARE-E has a small sample size and open label design, there are several strengths that differentiate CARE-E from other studies. The multicenter design allows for a wider range of study participants and prevents intrinsic bias in interpretation of study results. The recording of EEG activity in participants allows for an objective measurement of efficacy of the *Cannabis* herbal extract in relation to dosage and steady state pharmacokinetics. Procurement of external funding to perform this study also prevents perception of bias in the collection and reporting of study results.

## Abbreviations

CARE-E: Cannabidiol in Children with Refractory Epileptic Encephalopathy
CBD: Cannabidiol
EEG: Electroencephalogram
QoL: Quality of life
Δ^9^-THC: Δ^9^-tetrahydrocannabinol
